# Morphologic and Genomic Heterogeneity in the Evolution and Progression of Breast Cancer

**DOI:** 10.3390/cancers12040848

**Published:** 2020-03-31

**Authors:** Jamie R. Kutasovic, Amy E. McCart Reed, Anna Sokolova, Sunil R. Lakhani, Peter T. Simpson

**Affiliations:** 1 UQ Centre for Clinical Research, Faculty of Medicine, The University of Queensland, Herston, Brisbane 4029, Australia; j.kutasovic@uq.edu.au (J.R.K.); amy.reed@uq.edu.au (A.E.M.R.); Anna.Sokolova@health.qld.gov.au (A.S.); s.lakhani@uq.edu.au (S.R.L.); 2 QIMR Berghofer Medical Research Institute, Herston 4006, Australia; 3 Pathology Queensland, The Royal Brisbane & Women’s Hospital, Herston, Brisbane 4029, Australia

**Keywords:** breast cancer, genomics, intra-tumour heterogeneity, metastasis, subclonal diversity, treatment resistance

## Abstract

Breast cancer is a remarkably complex and diverse disease. Subtyping based on morphology, genomics, biomarkers and/or clinical parameters seeks to stratify optimal approaches for management, but it is clear that every breast cancer is fundamentally unique. Intra-tumour heterogeneity adds further complexity and impacts a patient’s response to neoadjuvant or adjuvant therapy. Here, we review some established and more recent evidence related to the complex nature of breast cancer evolution. We describe morphologic and genomic diversity as it arises spontaneously during the early stages of tumour evolution, and also in the context of treatment where the changing subclonal architecture of a tumour is driven by the inherent adaptability of tumour cells to evolve and resist the selective pressures of therapy.

## 1. Introduction

That breast cancer is heterogeneous is beyond all doubt. We now count at least 20 histological subtypes of invasive breast cancer, defined by morphologic growth patterns and cytological appearance [1] and three broad biological subtypes, based on the expression of diagnostic biomarkers (oestrogen (ER) and progesterone (PR) receptor positive; HER2 positive; and triple negative (lacking hormone receptors and HER2). The ‘big data’ revolution has dramatically enhanced our appreciation of the molecular heterogeneity of breast cancer, further stratifying the disease into biologically and clinically meaningful subtypes, including six or more intrinsic subtypes (normal, claudin-low, luminals A and B, HER2 enriched and basal) [2,3,4,5]; four triple negative molecular subtypes (basal-like 1, basal-like 2, mesenchymal and luminal androgen receptor) [6]; and, ten integrative clusters captured by combined transcriptional and DNA copy number profiling [7]. Adding to this, the diversity of extra-tumoral components such as the tumour matrix and immune infiltrate is substantial and so it is easy to imagine that no two breast cancers will respond to therapy, or potentially progress to metastasis in quite the same way.

The recent advances in genomics technology is providing elaborate detail to the somatic architecture of breast tumour genomes, and with it, unprecedented insight into the mechanisms at play driving tumour development, adaptation and progression in response to treatment. Next generation sequencing technology has built on foundational knowledge created by candidate gene sequencing and comparative genomic hybridisation to provide very high depth, targeted gene panel sequencing for identifying targetable mutations and subclonal mutations; whole exome sequencing (WES) for comprehensive mutational analysis of all coding sequences; and whole genome sequencing (WGS) for an unbiased survey of all coding and non-coding sequences to capture the full repertoire of genetic alterations, encompassing single nucleotide variants (SNVs), small insertions and deletions (indels), copy number alterations (CNAs) and structural variants (SVs). Most somatic genetic alterations are perceived to provide little or no advantage to the neoplastic cells in which they arise (passenger mutations), however some enhance or inhibit the activity of cancer genes, and hence are termed driver mutations. One of the great powers of WGS is the ability to use the large numbers of SNVs, indels, CNAs and SVs to call mutational signatures and the analysis of these ‘genomic scars’ reveal great insight into the causative factors driving an individual cancer [8,9,10,11,12]; i.e., the exogenous carcinogenic processes, defective endogenous cellular processes or germline predisposition that have played a significant role in the aetiology of an individual cancer.

Here we outline how the molecular genetic analysis of tumour genomes has shed light on the inter- and intra-tumour heterogeneity exhibited by breast cancer; we elaborate on the concepts of cancer drivers and clonal evolution linked directly to the diverse morphological characteristics of the disease; and the complex processes of metastasis.

## 2. Genomic Diversity of Primary Breast Cancer

There are several landmark studies that have characterised the genomic landscape of invasive breast cancers [10,11,13,14,15,16]. It is increasingly clear that each breast cancer is genomically distinct, with a high level of diversity in the overall number of individual genetic alterations (SNVs, indels, CNAs, SVs), the cancer genes affected, and the global patterns of mutations captured by mutational signatures. 

Most breast cancers have relatively low numbers of SNVs and indels, compared to other cancer types, however, approximately 20% of tumours are associated with defective homologous recombination (HR) double strand break repair (e.g., in particular those arising in *BRCA1*, *BRCA2*, *PALB2*, *RAD51*C germline mutation carriers), and these exhibit high rates of SNVs and indels. Further, a minority of tumours (<10%) exhibit hypermutator phenotypes, for instance in tumours associated with defective base excision repair (e.g., *MUTYH* inactivation), mismatch repair (e.g., *MSH2, PMS2*, *MLH1* inactivation) or APOBEC cytidine deaminase activity mutational signatures [8,9,12,14,17,18,19].

From an architectural point view, some breast cancers have ‘simple’ genomes (e.g., tumours with the 1q gain and 16q deletion pattern of alterations), whilst other tumours exhibit complex arrays of structural variants involving interchromosomal rearrangements and high level amplification of major oncogenic driver genes (e.g., including *ERBB2*/HER2, *CCND1*, *ZNF703*/*FGFR1*, *MYC*); and tumours associated with defective HR repair exhibit extremely high levels of chromosomal instability [7,9,13,14,20,21].

**Figure 1:**
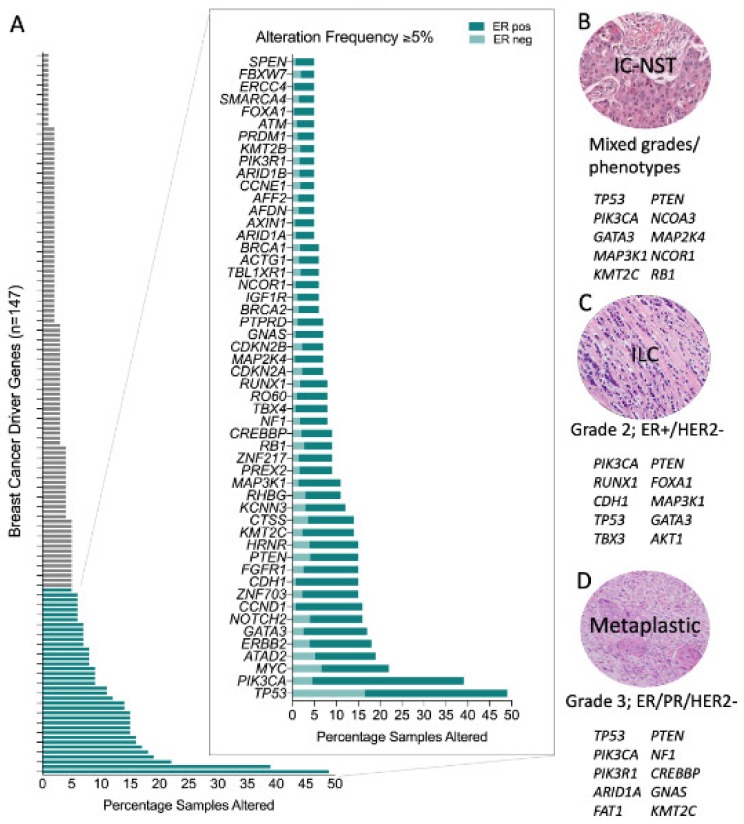
Genomic alterations across breast cancers. (**A**) Frequency of genomic alteration (mutation and copy number variation) in the 147 breast cancer driver genes across the TCGA pancancer breast cancer dataset (*n* = 1033); and stratified by oestrogen receptor (ER) status (in magnified plot): ER positive, *n* = 795; ER negative, *n* = 238. Top ten most frequently mutated genes in (**B**) Invasive Carcinoma-No Special Type (IC-NST) [15]; (**C**) Invasive Lobular Carcinoma (ILC) [15]; and, (**D**) Metaplastic breast cancer [36,37,38].

A meta-analysis of breast cancer sequencing studies has established that there are at least 147 breast cancer driver genes [22]. Approximately three driver gene mutations are found per tumour [14] and there are a multitude of combinations possible [23]. Some are mutated or altered at high frequency (e.g., *TP53*, *PIK3CA*, *MYC*, *CCND1*, *ERBB2*) whilst most are affected infrequently, with only 39/147 (26.5%) of these driver genes being altered in 5% or more of the TCGA breast cancer samples (Figure 1A). Further, some genes exhibit a strong genotype/phenotype relationship and so when altered they contribute to the resulting molecular and phenotypic lineage that subsequently develops. For instance, the distribution of driver mutations differs between ER positive and ER negative tumours [14], including the most common driver genes, *PIK3CA* and *TP53*, respectively. This is also evident in familial breast cancer, where the inheritance of a pathogenic germline driver mutation is also strongly related to the resulting tumour phenotype: ER-negative in *BRCA1*-associated tumours (with high frequency of TP53 mutations); ER-positive in *BRCA2*, *ATM* and *CHEK2-*associated tumours; HER2-positive in TP53-carriers; and E-cadherin negative and lobular growth pattern in *CDH1*-carriers [19,24,25,26,27,28,29].

Some driver mutations manifest more frequently in morphologically distinct tumours and some are pathognomonic for special histological types of the disease (Figure 1B–D). Elegant examples of this occur in rare breast cancer special types; for example secretory carcinomas arise due to the highly recurrent oncogenic driver created by a balanced t(12;15) (p13;q25) translocation creating an *ETV6-NTRK3* fusion gene; similarly the *MYB-NFIB* translocation (t(6;9) (q22–23; p23–24)) is a key driver in the development of adenoid cystic carcinomas of the breast. Both these tumour types are low-grade, typically of a triple negative phenotype and have counterparts in other tissues (e.g., salivary gland) driven by the same translocations [30,31,32,33,34].

Invasive lobular carcinoma (ILC) is the most common special histological type of breast cancer, defined by a characteristic diffuse growth pattern, with discohesive neoplastic cells. The archetypal alteration in ILC involves dysfunction of the epithelial cell adhesion complex involving E-cadherin and its binding partners β-catenin and P120-catenin. E-cadherin is encoded by the gene *CDH1*, which is inactivated in ~65% of ILC by gene mutation and loss of heterozygosity. Building on formative work by others, the recent large TCGA study [15], Desmedt et al. [35] defined the unique genomic features of ILC compared to invasive breast carcinoma of no special type (IBC-NST or IC NST, previously called invasive ductal carcinoma, IDC) through a deep characterisation of the TCGA breast cancer multi-omic data and the targeted mutation profiling of a large cohort of ILC. In addition to *CDH1* mutations, the only other highly recurrent oncogenic driver was *PI3KCA* (43–48%), with a plethora of low frequency (<15% of cases) driver mutations affecting *FOXA1*, *TBX3, ERBB2, ERBB3* and *PTEN* that were enriched in ILC relative to IC NST, while *GATA3* and *TP53* mutations were enriched in IC NST relative to ILC. TP53 mutations occur at significantly different frequencies between ER+ and ER− tumours, and so the *TP53* mutation finding is likely driven by the presence of ER negative tumours in the IC NST cohort. Metaplastic breast cancers are at the other end of the histological spectrum to ILC; they are a rare and heterogeneous special tumour type, which exhibit metaplastic change to squamous and/or mesenchymal elements; tumours are high grade and are associated with an overall poor outcome. Although generally triple-negative, they have a high frequency of *PIK3CA* mutations [36,37,38], and indeed have the unusual co-occurrence of *PIK3CA* and *TP53* driver mutations in some instances [36].

## 3. Subclonal Genomic Diversity in Primary Breast Cancer

**Figure 2:**
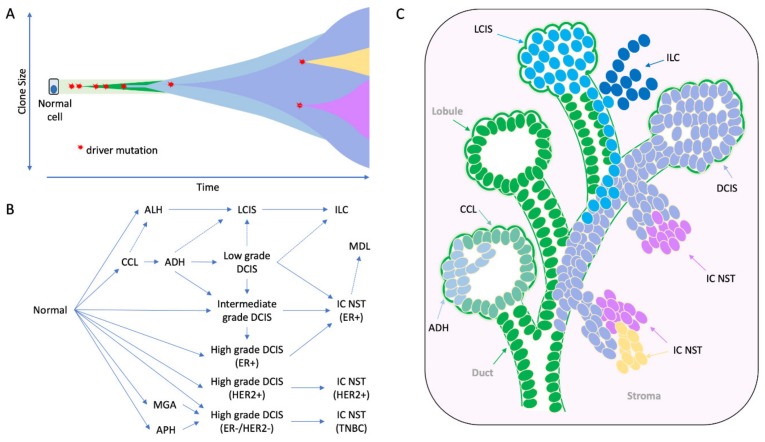
The morphological and molecular evolution of breast cancer. (**A**) Hypothetical schematic showing how the mutation of cancer genes drives the clonal and subclonal evolution of cancer (adapted from [55]). Key early driver genes impact the subsequent lineage and tumour type that arises, including mutations in *PI3KCA* in ER+ tumours, *CDH1* in lobular lineage, *TP53* in high grade ER- tumours, *ETV6-NTRK3* and *MYB-NFIB* translocations in secretory and adenoid cystic carcinomas respectively. (**B**) the multistep model of breast cancer showing morphological stages of development from normal epithelium. This simplified model is based on the evolution of ER positive and ER negative breast cancer, as portrayed in more detail elsewhere [49,50,51]; evidence derived from morphological evaluation and the frequency with which lesions are co-localized, as well as molecular evidence showing co-localized lesions share identical mutations indicating clonal relatedness. (**C**) Cartoon to illustrate how this might arise in a ‘sick lobe’, that is a clonal outgrowth of apparently morphologically normal-looking epithelial cells (green), which harbour early genetic changes. In some areas of the lobe, the earliest morphologically abnormal changes may appear in some terminal duct-lobular units (lobule) as columnar cell lesions. These lesions are considered precursors of ADH (light blue cells) and DCIS (purple cells), which arise in lobules and may travel down ducts. The mutation or loss of *CDH1* (E-cadherin) triggers the evolution of the ’lobular lineage’ (sky blue cells) as ALH then LCIS (lobular neoplasia); these cells may travel down ducts underneath the normal epithelial lining (pageotoid spread). Both LCIS and DCIS are genetically advanced lesions and so likely exhibit sub-clonal mutations. As these neoplastic cells can travel along ductal structures then this means invasion can occur at multiple sites giving rise to multifocal invasive disease (ILC, IC NST), which continues to undergo subclonal change. CCL: columnar cell lesion; ADH: atypical ductal hyperplasia. ALH: atypical lobular hyperplasia, APH: atypical apocrine hyperplasia; DCIS: ductal carcinoma in situ; LCIS: lobular carcinoma in situ; IC NST: invasive carcinoma no special type; ILC: invasive lobular carcinoma; MDL: mixed ductal lobular carcinoma; MGA: microglandular adenosis.

Multi-region sequencing of an individual tumour gives intriguing insights into the subclonal nature of the disease (Figure 2A). The level of subclonal heterogeneity identified across a cohort of 50 breast cancers was variable [39]: most cases had a driver mutation that was shared by all regions sequenced (i.e., an early founder driver gene mutation, and indicating an evolutionarily conserved lineage); about half the cancers showed limited variation in the mutations identified across different regions sequenced, whereas for three tumours there was profound subclonal diversity. Sub-clonal driver mutations (e.g., in *TP53*, *PIK3CA*, *PTEN*, *MYC* amplification) were identified in a subset of tumour regions sequenced. Subclonal driver alterations have been previously evident, but not to such detail, through more standard in situ techniques in the diagnostic setting, i.e., breast tumours with heterogenous *ERBB2* amplification. The geographical expansion of mutant subclones was often confined to 1–3 adjacent regions, but interestingly in some cases, mutationally distinct subclones were found to be growing admixed with one another. Cases studied pre- and post- neoadjuvant chemotherapy or targeted therapy revealed evidence that treatment can dramatically alter the clonal make-up of a tumour [39,40].

Eirew and colleagues [41] studied mutations and subclonal dynamics using patient-derived xenograft (PDX) models, and demonstrated that engraftment and subsequent propagation of patient samples led to selective changes in subclonal frequencies. Notably, independent grafts of the same tumour resulted in reproducible expansion of specific subclones that were presumably ‘fitter’ in this new environment [41]. These striking findings recapitulates the clonal diversity observed in patient samples, but also highlights the idea that human tumour cells in PDX models are dynamic and continually evolve in response to the pressures they are subjected to.

Mixed ductal lobular carcinomas are a unique histological subtype of breast cancer; like metaplastic breast cancers they elicit morphological evidence of intra-tumour heterogeneity, this time showing tumour regions with both ductal and lobular-like differentiation. Multi-region exome sequencing supplemented by copy number profiling of cases exhibiting distinct morphological components demonstrated these were clonally related tumour regions as opposed to being collision tumours [42]. In contrast to the above studies, where topographically defined regions were analysed, here morphologically defined populations of cells representing the different growth patterns (ductal and lobular, including associated pre-invasive lesions) were isolated by microdissection and analysed. In individual cases, all lesions shared precise genetic alterations as likely early events in tumour development; all cases also exhibited private mutations unique to a morphological lineage (e.g., *TBX3*), suggesting they may be important in the separate evolution from a common antecedent [42].

This theory is supported by data from an analysis of multiple invasive tumours from patients with multifocal breast cancer, using targeted gene sequencing analysis, supplemented by low coverage WGS to identify structural and copy number variants [43]. Here, all lesions within an individual case were morphologically identical and expressed the same biomarker profile (same grade, ER and HER2 status). In two thirds of cases, all lesions shared precise genetic alterations, whilst the remaining cases shared no common mutations, from the panel of 360 genes analysed, but they shared structural/copy number variants. Thus, all cases exhibited compelling evidence for the multifocal invasive tumours having a common clonal origin, and for there being subclonal, parallel/branched evolution occurring prior to invasion into the tissue stroma.

## 4. The Early Clonal Nature of Breast Cancer—Going Back to the Beginning

The early stages of breast neoplasia are defined by a plethora of morphologically characterised lesions which reside within the ductal tree. The frequency with which such morphologically distinct lesions co-existed in the same specimen gave credence to the idea that lesions were evolutionarily related (later supported by molecular evaluation). Ductal carcinoma in situ (DCIS) and lobular carcinoma in situ (LCIS) are genetically advanced lesions and direct precursors to invasive cancer. Columnar cell lesions (CCL), flat epithelial atypia (FEA), atypical ductal hyperplasia (ADH) and atypical lobular hyperplasia/lobular neoplasia (ALH/LN), among others, are considered ‘earlier’ steps along the multistep pathway to breast cancer development. Each of these lesions harbour genetic alterations and are considered clonal neoplastic proliferations; CCL harbour both DNA copy number alterations and gene mutations, including an usually high rate of *PIK3CA* mutations (54%) [44,45,46]. Likewise, ADH may be considered a genetically advanced precursor lesion [47].

**Figure 3:**
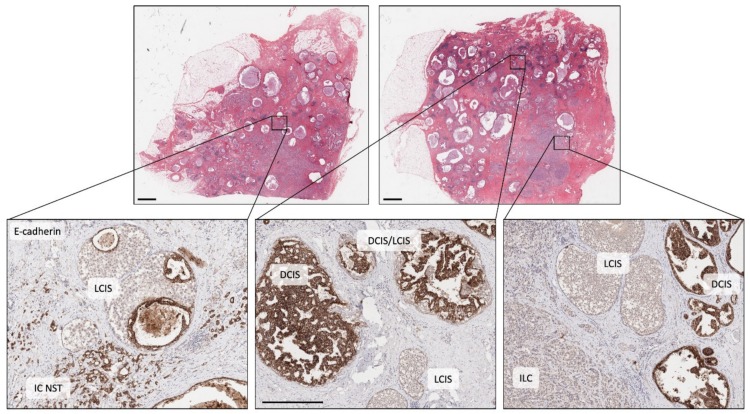
Illustrating morphological and molecular heterogeneity. Low power, haematoxylin and eosin stained sections of two tissue blocks from the same surgical specimen (scale bar = 2 mm). Both blocks are widely affected by breast disease with cystically dilated ducts, in situ carcinoma and invasive carcinoma. The three images in the lower panel are high power views of the same sections stained for E-cadherin (scale bar in middle image = 0.5 mm). Left picture shows cells of LCIS (E-cadherin negative) that have grown and then expanded underneath normal epithelial cells lining a duct (pagetoid spread), adjacent to E-cadherin positive, invasive cells of IC NST. Middle picture shows adjacent ducts in a complex branching network, one duct populated by DCIS (E-cadherin positive), two smaller ducts by LCIS and two other ducts co-involved by cells of DCIS and LCIS (DCIS/LCIS). Right picture showing ducts separately involved by DCIS or LCIS, plus an area of invasive cancer (ILC, E-cadherin negative). The individual components of this case were previously analysed by whole exome sequencing and all lesions were shown to be clonally related with early, common diver mutations identified in *BRCA2* and *TBX3*, ‘lobular’ lineage-specific mutations including in *CDH1* and ‘ductal’ lineage-specific mutations including in *NF1* (see [42]). DCIS: ductal carcinoma in situ; LCIS: lobular carcinoma in situ; IC NST: invasive carcinoma no special type; ILC: invasive lobular carcinoma.

The role these lesions play in the evolution of ER-positive and ER-negative disease types has been well described [34,48,49,50,51] (Figure 2B). Early hypotheses for the evolution of ER-positive breast cancer, in particular, was that of a linear progression from CCL to ADH to DCIS to IDC. Yet the level of intra-tumoural heterogeneity seen within precursor lesions of an individual specimen points to a more complex situation. For instance, within a surgical specimen both DCIS and LCIS can exhibit morphological (e.g., different grades/level of differentiation) and biological (e.g., variable expression of ER, PR, HER2, Ki67) heterogeneity as well as evidence of subclonal genomic diversity [52,53,54]; these lesions can also co-exist, even admixed within the same duct (Figure 2C and Figure 3). Whilst a linear process of evolution might occur, there is more likely a complex array of parallel/branching clones evolving within the normal ductal structure, and that this probably arises from an underlying bed of genetic instability already present in normal breast epithelium (Figure 2 and Figure 3).

Molecular analysis of morphologically complex cases has given great insight into this diversity. Topographically mapped single cell sequencing has elegantly demonstrated that most copy number alterations identified in invasive cancer arise in DCIS; and that clonal diversity observed in invasive cancer is driven in large part by existing clonal diversity present within DCIS, whereby distinct subclones may escape from the ductal tree to seed polyclonal invasive disease (neoplastic cells escaping from different regions of the ductal tree could seed apparently multifocal invasive cancer) [54,56] (Figure 2C). Weng and colleagues [57] also explored these relationships in detail using massively parallel sequencing of normal epithelium, various low-grade proliferative and pre-invasive lesions and associated invasive cancer. Using the somatic mutations to resolve phylogenetic relations between the lesions, the authors revealed a fascinating and complex hierarchy between lesions within individual cases; while IC NST and DCIS were always linked by a shared mutational history; CCLs were either (i) closely related to this DCIS/IC NST lineage with numerous shared somatic mutations, (ii) distantly related to this lineage owing to sharing very early mutations but subsequently evolving down a parallel pathway, or (iii) arose quite independently with no mutations detectable or no mutations in common to higher grade lesions analysed. Interestingly, *PIK3CA* mutations arise frequently but quite heterogeneously within this early stage of disease, including in normal epithelium. Sometimes these mutations are discordant between lesions examined, or are present in CCL but not in synchronous in situ and invasive lesions [45,57], suggesting such early driver events may enhance cellular proliferation, on a background of which other driver mutations may (or may not) arise to trigger progression (or not) to more advanced lesions.

Recent sequencing has revealed an amazing level of genetic instability in ‘normal’ cells of various tissues, caused by environmental exposure or local pathological processes related to tissue injury [58,59,60,61]. There is a growing wealth of evidence suggesting the same is true in breast tissue, acting as a primer for neoplasia. Indeed, morphologically normal epithelium adjacent to tumour harbours a higher level of genetic instability relative to reduction mammoplasty tissue, particularly when normal is within 1 cm of tumour; furthermore normal epithelium from cancer-free patients who carry a pathogenic germline mutation in *BRCA1* or *BRCA2* also acquire an elevated level of chromosomal instability compared to controls [57,62,63,64,65,66]. In the case of germline mutation carriers, haploinsufficiency for genes with clear roles in DNA damage response (such as *BRCA1* and *BRCA2*) is likely to underpin the predilection to acquire genomic alterations in cells prior to morphological abnormalities being observed [67,68,69]; in non-carriers the genetic instability may be arising as part of a field cancerisation or ‘sick lobe’ effect, in which a duct/lobe or a proportion of a lobe is clonally affected by genetic instability and hence the entire lobe is ‘at risk’ of further genetic instability and oncogenic activation [70]. Indeed, this might explain the observation of multiple atypical proliferations (e.g., CCL, ADH, LCIS, DCIS) co-existing across the same specimen (Figure 2C and Figure 3).

## 5. Genomics and Clonal Dynamic Changes During Metastatic Progression

Metastatic dissemination is the cause of most cancer-related deaths, therefore, the goal to develop a deep understanding of the mechanisms of metastasis cannot be understated. Large scale sequencing projects of metastatic samples from breast cancer patients, and the analysis of matched cases of the primary tumour and distant metastasis or multiple metastases from an individual have started to reveal important advances in knowledge of clonal progression and treatment resistance.

In many cases the growth pattern (histological type), the expression of phenotypic biomarkers and the molecular subtype of the primary tumour remains quite stable during progression of disease. Genomic data reveal a high concordance in the mutations and in particular copy number alterations between matched primary and metastatic tumours [71,72,73,74,75]. Thus, there is a clear clonal ancestry during progression, and the early molecular drivers of behaviour and phenotype (e.g., mutations in *TP53, PIK3CA*, *CDH1*, *GATA3*, amplification of *MYC, CCND1, ERRB2*/HER2) remain prevalent drivers in metastatic deposits [71,72,73,74,76,77,78,79,80]. Despite this, significant intra-patient heterogeneity develops during progression, even in the absence of systemic therapy; this occurs to a greater extent in progression to distant metastases relative to local lymph nodes and is exacerbated by the selective pressures applied during adjuvant therapy [73,77,81,82,83,84]. Changes in tumour phenotype or in the intrinsic molecular subtype during progression occurs in around 30% of patients (most often involving the down regulation of PR, but may also involve ER and less frequently a change in HER2 status) and may occur in a non-random manner at specific metastatic sites (e.g., lung, liver and bone metastases) [85,86,87,88]. To further complicate matters, the phenotype of different metastases within a patient can be heterogenous [83,86,89,90,91,92].

Compared to early breast cancer, distant metastases tend to harbour a higher mutation burden and more frequent alterations to driver genes that may confer resistance to chemotherapy or targeted therapy, in particular endocrine therapy [74,75,78,79,80]. Most notably, activating mutations in *ESR1* and amplification of the *ESR1* gene region (6q25.1) are rarely observed in primary disease, but are prominent and critical drivers of resistance observed in around 20% of metastases arising following endocrine therapy [73,74,78,79,80,93,94]. Enrichment of mutations in *TP53*, *GATA3*, *KMT2C*, *AKT1*, *NF1*, *PTEN*, *ERBB2*, *FGFR4*, or amplification of 7p11.2 (*EGFR*), 8q24 (*MYC*), 11q13.3 (*CCND1*) and 20q13.2 (*AURKA*) may also underpin endocrine therapy resistance as they are more frequently identified in ER+/HER2− breast cancer metastases compared to ER+/HER2− primary tumours; many of these gene mutations are mutually exclusive to *ESR1* mutations, emphasising their potential equivalence in driving resistance [78,79,80,95].

Mutational signatures found in the primary tumour are also found in metastases; but as with individual gene mutations, the frequencies of individual mutation signatures may also change, with an enrichment in signatures associated with APOBEC enzymatic activity and homologous recombination deficiency being higher in metastases than in primary tumours [75,78,82]. Evidence suggests the acquisition of APOBEC signature maybe a driver of intra tumour heterogeneity and endocrine resistance [17,84,96,97].

The genomic analysis of matched primary and metastatic samples has revealed fascinating insight regarding the evolution of metastatic disease [73,75,82,83,84,86,90,98,99,100]. Such efforts reveal, for example, that driver mutations that are enriched in metastasis are indeed rarely found in the matched primary tumour, indicating they arose either in a small subclone not sampled when the primary tumour was sequenced, or they occurred during the metastatic process after cells had disseminated from the breast (i.e., treatment induced mutations) [39,73,79,80,82,101]. Indeed, mutations in *ESR1*, *ERBB2* and *NF1* were significantly enriched in ER+/HER2− tumours post hormone treatment compared to tumours from ER+/HER2− untreated patients [80].

The genetic relationship between multiple metastases within a patient is exceedingly complex but accruing sequencing data and phylogenetic analysis suggests that all metastases within a patient are genetically related, arising from a common ancestral clone. However, subclonal divergence of metastases is invariably observed within patients: driver and non-driver gene mutations are heterogeneously accumulated in different metastases, subsets of metastases may therefore be more closely related to each other than they are to other metastases, and heterogenous tumour phenotypes (ER positive and ER negative) often coincide with this divergent history [75,84,90].

The data supports various models of progression; evidence for both linear and parallel models are evident, in which multiple metastases may arise from a single seeding event from cells disseminating from the primary tumour, or indeed metastases may be seeded from already established metastases in a more linear fashion. The longer the time span between diagnosis of primary tumour and that of the metastases, then the larger the divergence in genetic make-up of the metastases, as expected [98]. Further, in patients with advanced disease at the time of diagnosis, there is evidence of multiple seeding events from the primary tumour, or even from different parts of the same primary tumour [84,98].

An important finding arose through the analysis of the variant allele frequency of shared mutations between metastases or between the primary tumour and resulting metastases: subclonal mutations remained subclonal in the resulting tumour, indicating that metastases were seeded by heterogeneous collections of disseminated cells as opposed to being seeded by a single cell or a single clone (monoclonal origin) [75].

## 6. Capturing Intra-Tumour Heterogeneity in Tissue or Liquid Biopsy

Predicting the extent of intra-tumour heterogeneity in primary or metastatic disease may provide valuable diagnostic insight into improving the management of patients undergoing neoadjuvant or adjuvant therapy, respectively. This may provide a framework for understanding likely response to chemotherapy or targeted therapy in these settings. As described above, it is clear that tumours may develop and progress on a linear, monoclonal trajectory, with little diversity in phenotype. A single biopsy of the primary tumour or a metastatic deposit may therefore be sufficient to capture the most functionally important alterations to determine therapy.

However, tumours that exhibit intra-tumour heterogeneity and hence with parallel/branching models of progression at play, are more likely to harbour subclones with innate treatment resistance or metastatic capability, or to harbour the capability to evolve in response to treatment to develop resistance. Capturing this level of intra-tumour heterogeneity at diagnosis maybe challenging, but could encompass the recording of a heterogeneity score with regards to morphology and biomarker expression/molecular subtype. Pathologists already record the presence of mixed growth patterns or grades, or the diversity across a tumour for the expression of ER, PR, HER2. Comprehensive sequencing of the entire primary tumour to characterise the subclonal architecture of a mass is not feasible, but evidence suggests that sequencing of two different regions of the tumour provides meaningful information to record clonal heterogeneity and to identify targetable genomic alterations [40].

There has been some reluctance to biopsy metastatic disease in the past, but it has great value in the era of molecular evaluation and the potential offerings for precision medicine. Various studies have demonstrated the feasibility in performing molecular testing on metastatic biopsies [71,79], but this approach is only possible if the metastasis is accessible and may not be appropriate when a patient has multiple organs involved.

Alternative approaches to examine tumour heterogeneity or for capturing important phenotypic or genomic alterations have advanced significantly in recent years. Circulating tumour cells (CTCs) and cell-free tumour DNA (ctDNA) [102,103,104] are shed into the circulation from both primary and metastatic tumour deposits. Such liquid biopsies are very accessible, and very amenable to repeat sampling while the patient is on treatment to monitor disease. They are, therefore, of great potential benefit in capturing phenotypic heterogeneity or driver mutations acquired or enriched for during treatment; and they are not biased by tumour sampling.

Increased concentration of CTCs in early [105,106] and metastatic [107,108,109,110] breast cancer is associated with poor prognosis. The application of single cell analysis technologies to PDX models has shown that CTCs are continuously released by the primary tumour, however only a proportion of clones have the capacity to seed a metastatic deposit, and as such the utility of CTCs in predicting the characteristics of subsequent metastases may be limited [111]. Nevertheless, analysis of CTCs can capture phenotypic heterogeneity of the tumour of origin, for example in the expression of ER, HER2 and androgen receptors [112,113,114], and also of biological processes driving metastasis such as dynamic changes in epithelial and mesenchymal composition [115,116]. Clusters of CTCs, which may show intermediate epithelial/mesenchymal properties [115,117], demonstrate higher metastatic capacity than single cells [118,119,120,121]. Genomic analysis of single CTCs reveals important heterogeneity in the mutation of various driver genes (e.g., *PIK3CA, ESR1*, *KRAS, PTCH1, NOTCH1*) reflecting the presence of discrete subclonal mutations within the tumour of origin and/or the presence of genomic alterations driving resistance/metastasis [122,123,124,125,126,127].

To illustrate clinical utility of the serial evaluation of molecular heterogeneity within single CTCs, Paoletti and colleagues comprehensively profiled single CTCs in a patient with metastatic lobular carcinoma who progressed following chemotherapy [124]. They demonstrated the presence of four alterations (*CDH1* and *TP53* frameshift mutations; *PIK3CA* and *SOX2* amplifications) in CTC samples at baseline and progression. However, high-level *MYCN* amplifications were only identified in CTCs sampled at progression, likely conferring treatment resistance. Similarly, the development of mutations and splice variants within *ESR1* identified in single CTCs of metastatic patients on endocrine therapy also correlated with the onset of endocrine resistance [124,126,128,129].

*ESR1* mutations are also readily detected in ctDNA [130,131,132,133], and in fact, ctDNA represents a more sensitive method of detection compared to CTCs [129]. ctDNA is released from tumour cells undergoing apoptosis, necrosis and phagocytosis. Like CTC analysis, ctDNA provides an opportunity for non-invasive molecular testing, akin to the non-invasive prenatal testing (NIPT) in pregnancy, for monitoring patients on therapy. In early breast cancer, the detection of ctDNA in patients undergoing neoadjuvant chemotherapy correlated with tumour grade and stage and a slow (versus rapid) drop of ctDNA levels after one cycle of chemotherapy was associated with a shorter disease-free survival [134]. The detection of minimal residual disease was also demonstrated in patients who continued to have detectable ctDNA *PIK3CA* mutations after surgery [135]. In the metastatic setting, the mutational status is highly concordant between ctDNA and tumour tissue [136,137] with additional private mutations identified in some cases [137].

Serial ctDNA mutation analysis can help characterise the dynamic evolution of subclonal mutations in real time [138,139,140] and hence represents a powerful approach for the prospective analysis of patients on targeted therapy and the early detection of tumour subclones with resistance capability. This has been demonstrated in the setting of endocrine therapy (various types of *ESR1* alterations, including mutations, rearrangements and amplifications), CDK4/6 inhibition with endocrine therapy (*ESR1*, *RB1* and *PIK3CA* mutations) and anti-HER2 therapy (copy number variations in the *ERBB2* gene as well as increase in *TP53* or PI3K/AKT/mTOR pathway mutations) [131,132,141,142]. Importantly, and reflecting the inter-metastasis molecular heterogeneity described above, *ESR1* mutations identified from either CTC or ctDNA from an individual patient are often heterogenous, suggesting that distinct subclones develop in parallel and utilise overlapping mechanisms of resistance [124,131].

## 7. Clinical Implications and Utility in Breast Cancer

Many major centres around the world operate routine cancer sequencing programs integrating clinical applications with research, and commonly using targeted panels of cancer genes [143] for triaging patients into clinical trials for targeted therapies. For example; Dana Farber Cancer Centre /Brigham and Womens’ Cancer Centre (BWCC) offers the ‘Profile’ study wherein cancer gene panel testing may help doctors enrol a patient in a clinical trial or choose the right combination of FDA-approved targeted therapies. Memorial Sloan Kettering Cancer Centre has been pioneering ‘basket trials’ implementing the use of their in-house MSK-IMPACT panel sequencing assay [144], where trial inclusion is based on mutation status rather than disease origin. UC San Diego Moores Cancer Centre uses the Foundation One panel and has matched 45% of BC patients to a ‘personalised’ therapy [145,146], however it should be noted that most of these matches were *ERBB2* amplifications to HER2 therapies and the applicability of this panel outside of *ERBB2* in breast cancer is uncertain. Increasing numbers of tools are emerging to facilitate the matching of alterations and therapies, including for example, PanDrugs [147], while the MD Anderson program [148] is feeding back ‘sequence-drug’ matching data into the public arena through their Precision Cancer Therapy interface.

By the end of 2015, 39 gene targets with matched FDA-approved therapies were noted in an extensive review of precision oncology [149] while the OncoKB resource [150] details 20 genes (42 alterations) as FDA-recognised biomarkers (Level 1 evidence) and 10 genes (22 alterations) as Level 2 (standard of care; predictive of response in breast cancer or another indication). In breast cancer, *ERBB2* amplifications (targeted with anti-HER2 therapies) and *PIK3CA* mutations (targeted with Alpelisib + Fulvestrant) are the only Level 1 biomarkers as noted by OncoKB, while inactivating mutations of *BRCA1* and *BRCA2* are classed as Level 2 biomarkers for intervention with talazoparib and olaparib. Increasing data therefore supports the clinical application of genomics to inform therapeutic intervention in breast cancer. Whole exome and whole genome sequencing will be required to account for the diversity of genes mutated in breast cancer [14,151] as well as larger scale alterations and mutation signatures that may predict treatment response. It is now clear, through mutation signature analysis, that hallmarks of defective DNA damage repair (specifically homologous recombination which BRCA1/2 mediate) are indicative of dysfunctional *BRCA1*/*2* [8,10]. A weighted model (HRDetect) can detect *BRCA1/BRCA2*-deficient samples using WGS data [9]. The HRDetect algorithm was independently validated, and its association with platinum response in advanced breast cancer demonstrated, where a high HRDetect score was associated with clinical improvement on platinum therapies [152,153]. Recent research has applied a functional HR assay (RECAP) to breast cancer samples and demonstrated that 29% of HR-defective tumours were not *BRCA*-related [154], although the researchers themselves classify this approach as pseudo-diagnostic. 

The introduction of immune checkpoint inhibitors (ICI) has revolutionised therapeutics across a number of advanced solid tumours. While a subset of patients displays a durable response, the implementation of a robust biomarker has been challenging. Tumour mutation burden (TMB) is now emerging as a diagnostic biomarker for ICIs such as PD-1/PD-L1 inhibitors [155,156]. It is now possible to calculate TMB from panel sequencing data [157], not just exome or genome sequencing, and with time we expect to see a rationalisation of diagnostic ‘cut-offs’. Small molecular inhibitors of the PI3K/AKT/mTOR pathway are fast approaching the clinic. The pan-AKT inhibitor, AZD5363, is potent and sensitivity is predicted by *PIK3CA* mutations [158]. The SOLAR trial [159] investigating the mutant PIK3CA inhibitor, alpelisib, demonstrated that a combination of alpelisib with fulvestrant prolonged progression-free survival among patients with *PIK3CA*-mutated, HR-positive, HER2-negative advanced breast cancer.

The application of these genotype–phenotype relationships in the clinical context of heterogeneity remains to be rationalised. Tumour heterogeneity exclusive of a histological subtype is not standardly reported; for example, ER positivity is recorded in a binary fashion with a low cut-off for positivity. Whether drugs are used sequentially to target residual clones, or in combination for simultaneous targeting will depend on myriad factors including the application of robust biomarkers of sensitivity to the therapy and the extent of intra-tumour heterogeneity.
